# Supplementary material for: Multiscale modeling of blood circulation with cerebral autoregulation and network pathway analysis for hemodynamic redistribution in the vascular network with anatomical variations and stenosis conditions
Source: PLoS Comput Biol. 2026 May 18;22(5):e1013853. doi: 10.1371/journal.pcbi.1013853 (PMC13211260; doi:10.1371/journal.pcbi.1013853)
Supplement: S1 Text — (PDF) [file pcbi.1013853.s001.pdf]

S1 Text. Pseudocode of the proposed CAM-incorporated path-flow inversion framework

This document provides pseudocode for the algorithm described in Section 2 of the manuscript (Fig 2). The solver consists of three nested levels: an *outer loop* that couples the 0D resistance network with the 1D blood flow model (Algorithm 2), a *middle loop* that performs CAM (Algorithm 3), and an *inner time-stepping* loop for the 1D Lax–Friedrichs scheme (Algorithm 4). After Stage 1 converges, the path-flow inversion (Stage 2) decomposes the segment-level flows into source-to-sink contributions (Algorithm 5).

Network specification

The cerebral arterial network contains $N_{\text{line}} = 130$ vessel segments and $N_{\text{node}} = 118$ nodes, reconstructed from CT images (Ii et al. 2020). Four inflow sources (RICA, LICA, RVA, LVA) connect the heart model to the cerebral circulation, and 45 outflow sinks represent terminal arterial endpoints grouped into six vascular territories (RACA, LACA, RMCA, LMCA, RPCA, LPCA). All 130 vessel segments are modelled with both 0D (resistance) and 1D (Lax–Friedrichs) representations; the extended cardio-cerebral coupling adds 7 extra resistive elements (R_{aorta}, R_{up}, R_{arm}, R_{body}) that are treated as 0D only (Fig 1c).

1 Stage 1: CAM-Incorporated 0D–1D Multiscale Model

Algorithm 1: Initialization

Input: Vessel geometry from ScanIPData (lengths L_{ij}, radii r_{ij}); cardiac pressure waveform $P_{\text{heart}}(t)$; pathological condition $c \in \{\text{Baseline, PCA, ACA}\}$; cardio-cerebral coupling parameters: R_{aorta}, $R_{\text{up},i}$, R_{arm}, R_{body}; CAM parameters: $\bar{V}_{\text{sa}}$, $\bar{R}_{\text{sa}}$, $\bar{C}_a$, ΔC_a^+, ΔC_a^-, G_q per territory.

Parameters: $\gamma = 0.5$, $\varepsilon_{\text{cam}} = 5\times10^{-3}$, $\varepsilon_{\text{outer}} = 0.01$, $\alpha_{\text{relax}} = 0.3$, $\mu = 0.0045\,\text{Pa s}$, $\rho = 1050\,\text{kg/m}^3$.

Output: Converged $Q(x,t)$, $A(x,t)$ for all vessels.

`/* Construct 0D resistance matrix from geometry, Eq. (1) */`

for *vessel* ij, $i = 1, \ldots, N_{\text{line}}$ **do**
- $L_{ij} \leftarrow$ sum of centreline segment distances;
- $r_{ij} \leftarrow$ mean radius;
- $R^0_{ij} \leftarrow 8\mu L_{ij}/(\pi\, r^4_{ij})$;

`/* Build extended cardio-cerebral network (Fig 1c) */`

Add nodes: Heart, Aorta, Right arm, Body and left arm;

Connect: Heart $\to$ Aorta(R_{aorta}) $\xrightarrow{R_{\text{up},i}}$ cerebral inlets;

Aorta $\xrightarrow{R_{\text{arm}}}$ Right arm, Aorta $\xrightarrow{R_{\text{body}}}$ Body + left arm;

`/* Apply pathological condition */`

if *PCA* **then** $R_{\text{line}\,24} \leftarrow R_{\text{line}\,24} \times 10^8$ (occlude P1 segment);

if *ACA* **then** $R_{\text{line}\,21} \leftarrow R_{\text{line}\,21} \times 10^8$ (occlude A1 segment);

Algorithm 2: Outer Loop: 0D–1D Coupling

for $n = 1, 2, \ldots, N_{\text{outer}}^{\max}$ (default 20) **do**

`/* Middle loop: CAM (Algorithm 3) */`

$(\mathbf{Q}_{0\text{D}}, \mathbf{P}) \leftarrow \textsc{SolveCAM}(\mathbf{R}_{\text{current}})$;

`/* Solve 1D blood flow equations, Eq. (8) */`

$(\mathbf{Q}_{1\text{D}}^{n}, \mathbf{A}_{1\text{D}}^{n}) \leftarrow \textsc{Solve1D}(\mathbf{Q}_{0\text{D}}, \mathbf{P})$;

`/* Update resistance from 1D area, Eq. (9) */`

for *cerebral vessel* ij, $i = 1, \ldots, N_{\text{line}}$ **do**

$$R_{ij}^{\text{eff}} \leftarrow \frac{1}{N_t} \sum_t \sum_k \frac{\pi^2 \Delta x}{A_k(t)^2} C_R \qquad \text{(spatial integral, Eq. 9)};$$

$$R_{ij}^{(n+1)} \leftarrow (1-\gamma) R_{ij}^{(n)} + \gamma R_{ij}^{\text{eff}};$$

`/* Outer convergence check, Eq. (10) */`

if $n > 1$ **then**

$$J_n \leftarrow \frac{1}{N} \sum_{w=1}^{N} \left[\frac{\max_t |Q_w^n - Q_w^{n-1}|}{\max_t |Q_w^{n-1}|} + \frac{\max_t |A_w^n - A_w^{n-1}|}{\max_t |A_w^{n-1}|} \right];$$

if $J_n < \varepsilon_{\text{outer}}$ **then break** (converged);

$\mathbf{Q}_{\text{prev}} \leftarrow \mathbf{Q}_{1\text{D}}^{n}$; $\mathbf{A}_{\text{prev}} \leftarrow \mathbf{A}_{1\text{D}}^{n}$; $\mathbf{R}_{\text{current}} \leftarrow \mathbf{R}_{\text{new}}$;

Algorithm 3: Middle Loop: CAM

Input: $\mathbf{R}_{\text{current}}$ (resistance matrix for current outer cycle).

Output: Converged $\mathbf{Q}_{0\text{D}}$, $\mathbf{P}$ (flow and pressure over one cardiac cycle).

Initialize $V_{\text{sa},m} = 1$, $f_m = 1$ for $m = 1, \ldots, 6$; $P_{1,m}^{\text{prev}} = \bar{P}_{1,m}$;

Allocate rolling buffer $\mathbf{q}_{\text{hist}}[6 \times 7]$;

Solve initial 0D network: $\mathbf{P}$, $\mathbf{Q} \leftarrow$ **Pressuresolution**, **ComputeQ**;

for $k = 1, 2, \ldots, k_{\max}$ (default 500) **do**

for $m = 1, \ldots, 6$ **do**

(a) $q_m \leftarrow \text{mean}(|Q_{\text{territory}}^{(m)}|)$; $\delta q \leftarrow (q_m - \bar{q}_m)/\bar{q}_m$;

(b) Asymmetric sigmoid compliance (Eq. 5);

$$\Delta C_a \leftarrow \begin{cases} \Delta C_a^{+} & \text{if } q_m \le \bar{q}_m \\ \Delta C_a^{-} & \text{otherwise} \end{cases};$$

$$C_{a,m} \leftarrow \bar{C}_{a,m} + \tfrac{1}{2} \Delta C_a \tanh\!\big(-2 G_{q,m}\, \delta q / \Delta C_a\big);$$

(c) Volume update: $V_{\text{sa},m} \leftarrow V_{\text{sa},m} + C_{a,m} (P_{1,m} - P_{1,m}^{\text{prev}}) / \bar{R}_{\text{sa},m}$;

$P_{1,m}^{\text{prev}} \leftarrow P_{1,m}$;

(d) Factor update with relaxation;

$f_m \leftarrow f_m + \alpha_{\text{relax}} (V_{\text{sa},m}^{-2} - f_m)$;

(e) Re-solve full 0D network with updated f_m;

`/* Convergence check, Eq. (6) */`

$\mathbf{q}_{\text{now}} \leftarrow [q_1, \ldots, q_6]^{\top}$; $\text{col} \leftarrow \text{mod}(k-1, 7) + 1$;

if $k > 7$ **then**

$\Delta q_{\max} \leftarrow \max |\mathbf{q}_{\text{now}} - \mathbf{q}_{\text{hist}}(:, \text{col})|$;

if $\Delta q_{\max} < \varepsilon_{\text{cam}}\, \text{mean}(\bar{\mathbf{q}})$ **then break**;

$\mathbf{q}_{\text{hist}}(:, \text{col}) \leftarrow \mathbf{q}_{\text{now}}$;

Algorithm 4: 1D Blood Flow Solver (Lax–Friedrichs)

Input: 0D flow waveform $Q_{\mathrm{0D}}(t)$, vessel geometry (r_0, L), wall parameters (E_s, h_0, K_R).
Output: Q_{1D}, A_{1D}; per-cell area $A_k(t)$ for resistance integral.

for *vessel* $iv = 1, \ldots, N_{\mathrm{line}}$ **do**
  $A_0 \leftarrow \pi r_0^2$; $\Delta x \leftarrow L/N_{\mathrm{cell}}$; $c \leftarrow \sqrt{E_s h_0/(2\rho r_0)}$;
  $K_p \leftarrow c^2/A_0$; Sub-steps: $N_{\mathrm{sub}} = \lceil \Delta t_{\mathrm{0D}}/(C_{\mathrm{CFL}}\,\Delta x/c) \rceil$;
  Initialize $A_i = A_0$, $Q_i = Q_{\mathrm{0D}}(t_0)$ for $i = 1, \ldots, N_{\mathrm{cell}}$;
  for *each 0D interval* $[t_n, t_{n+1}]$ **do**
    for $s = 1, \ldots, N_{\mathrm{sub}}$ **do**
      $Q_1 \leftarrow$ interpolated inlet flow;
      for $i = 2, \ldots, N_{\mathrm{cell}} - 1$ **do**
        $A_i^{n+1} = \frac{1}{2}(A_{i-1} + A_{i+1}) - \frac{\Delta t}{2\Delta x}(Q_{i+1} - Q_{i-1})$;
        $F_L = A_{i-1}\, K_p\,(A_{i-1} - A_{0,i-1})$; $F_R = A_{i+1}\, K_p\,(A_{i+1} - A_{0,i+1})$;
        $Q_i^{n+1} = \left[\frac{1}{2}(Q_{i-1} + Q_{i+1}) - \frac{\Delta t}{2\Delta x}(F_R - F_L)\right](1 - K_R\,\Delta t)$;
      Outlet: $Q_{N_{\mathrm{cell}}} = Q_{N_{\mathrm{cell}}-1}$, $A_{N_{\mathrm{cell}}} = A_{N_{\mathrm{cell}}-1}$;

2 Stage 2: DFS-Based Path-Flow Inversion

While Stage 1 determines the flow rate and direction in every vessel segment, it does not directly answer the question: for a given terminal territory (e.g. RMCA), what fraction of its perfusion originates from each inflow source (RICA, LICA, RVA, LVA)? The path-flow inversion decomposes the converged segment flows into source-to-sink contributions using the formulation in Section 2.3 of the manuscript (Eqs. 13–16). This is an algebraic post-processing step based on flow conservation.

Algorithm 5: Path-Flow Inversion and Source Attribution (Eqs. 13–16)

Input: Network topology, converged segment flows $\bar{\mathbf{Q}}$, resistance matrix $\mathbf{R}$, territory definitions.
Parameters: δ (L1 sparsity), σ (energy), λ_1, λ_2.
Output: Source-to-sink attribution $\theta_{i,j}$; per-territory flow matrix barValues $[4 \times 65]$.

`/* Step 1: Enumerate candidate paths */`
for *source* $i \in \{$*RICA, LICA, RVA, LVA*$\}$, *sink* j **do**
  Find all possible paths via DFS;
Filter: keep only paths whose traversal direction matches $\mathrm{sign}(\bar{Q}_l)$ on every segment l;

`/* Step 2: Solve sparse path-flow inversion, Eq. (15) */`
Build $\mathbf{W}$, $\mathbf{b}$ from Eq. (14);
$\min_{\mathbf{x}\geq 0} \ \frac{1}{2}\|\mathbf{W}\mathbf{x} - \mathbf{b}\|^2 + \delta\,\|\mathbf{x}\|_1 + \sigma \sum_p R_p\, x_p^2$ (solved via CVX / SDPT3);

`/* Step 3: Source-to-sink attribution, Eq. (16) */`
for *source* i, *sink* j **do**
  $\theta_{i,j} \leftarrow \dfrac{\sum_{p:\,\mathrm{start}(p)=i,\,\mathrm{end}(p)=j} x_p}{\sum_{p:\,\mathrm{end}(p)=j} x_p}$;

`/* Step 4: Territory aggregation */`
Aggregate θ and $\mathbf{x}$ into six territories (RACA, LACA, RMCA, LMCA, RPCA, LPCA);
