# Supplementary material for: Multiscale modeling of blood circulation with cerebral autoregulation and network pathway analysis for hemodynamic redistribution in the vascular network with anatomical variations and stenosis conditions
Source: PLoS Comput Biol. 2026 May 18;22(5):e1013853. doi: 10.1371/journal.pcbi.1013853 (PMC13211260; doi:10.1371/journal.pcbi.1013853)
Supplement: S2 Text — (PDF) [file pcbi.1013853.s002.pdf]

S2 Text. Parameter values for the heart model and CAM

In the 0D heart model, $e(t)$ signifies the normalized time-varying elastance, which for the atria is

$$e_a(t)=\begin{cases}\frac{1}{2}\left\{1+\cos\left[\pi(t+\tau-t_{ar})/\tau_{arp}\right]\right\} & 0\le t\le t_{ar}+\tau_{arp}-\tau,\\ 0 & t_{ar}+\tau_{arp}-\tau<t\le t_{ac},\\ \frac{1}{2}\left\{1-\cos\left[\pi(t-t_{ac})/\tau_{acp}\right]\right\} & t_{ac}<t\le t_{ac}+\tau_{acp},\\ \frac{1}{2}\left\{1+\cos\left[\pi(t-t_{ar})/\tau_{arp}\right]\right\} & t_{ac}+\tau_{acp}<t\le\tau,\end{cases} \tag{S2.1}$$

and for ventricles is

$$e_v(t)=\begin{cases}\frac{1}{2}\left[1-\cos(\pi t/\tau_{vcp})\right] & 0\le t\le\tau_{vcp},\\ \frac{1}{2}\left\{1+\cos\left[\pi(t-\tau_{vcp})/\tau_{vrp}\right]\right\} & \tau_{vcp}<t\le\tau_{vcp}+\tau_{vrp},\\ 0 & \tau_{vcp}+\tau_{vrp}<t\le\tau.\end{cases} \tag{S2.2}$$

Here, τ represents the cardiac cycle duration. Furthermore, τ_{acp}, τ_{vcp}, τ_{arp}, and τ_{vrp} respectively represent the duration of atrial/ventricular contraction/relaxation. Also, t_{ac} and t_{ar} respectively denote the time points in one cardiac cycle at which the atria begin to contract and relax. The corresponding parameter values for the atrial and ventricular elastance functions are summarized in Table A.

Table A. Parameter values for the heart model (Liang et al. 2009).

Parameter	Right atrium (*ra*)	Right ventricle (*rv*)	Left atrium (*la*)	Left ventricle (*lv*)
$E_{cm,A}$ (mmHg/mL)	0.06	0.55	0.07	2.75
$E_{cm,B}$ (mmHg/mL)	0.07	0.05	0.09	0.08
τ_{cp} (s)	0.17	0.30	0.17	0.30
τ_{rp} (s)	0.17	0.15	0.17	0.15
t_c (s)	0.8	0.0	0.80	0.0
t_r (s)	0.97	0.30	0.97	0.30
S_v (mmHg s/mL)	$P_{ra}\times0.0005$	$P_{rv}\times0.0005$	$P_{la}\times0.0005$	$P_{lv}\times0.0005$

For the CAM, parameters used to define the autoregulatory part of the cerebral network were provided by Payne (2006) and McConnell and Payne (2017). As shown in Figs 1 and 2, each terminal node can be attached to a major cerebral artery in CoW, i.e., the left (L) and right (R) anterior cerebral arteries (ACAs), middle cerebral arteries (MCAs), and posterior cerebral arteries (PCAs). Therefore, this study sets that all vessels originating from the same major cerebral artery share identical arteriolar properties, as summarized in Table B.

Table B. Parameter values for the CAM (Payne 2006; McConnell and Payne 2017).

Parameter	$V_{sa,0}$ mL	G_q mL/mmHg	$C_{a,0}$ mL/mmHg	ΔC^+_a mL/mmHg	ΔC^-_a mL/mmHg	$R_{sa,0}$ mmHg s/mL	R_v mmHg s/mL
MCAs	4.71	1.18	0.08	1.13	0.06	32.60	12.18
PCAs	3.46	0.87	0.06	0.83	0.05	60.50	22.61
ACAs	3.96	0.99	0.07	0.95	0.05	46.31	17.30
Parameter	P_h mmHg	P_v mmHg	P_{ic} mmHg				
Whole network	108	5	10	-	-	-	
