# Supplementary material for: Multiscale modeling of blood circulation with cerebral autoregulation and network pathway analysis for hemodynamic redistribution in the vascular network with anatomical variations and stenosis conditions
Source: PLoS Comput Biol. 2026 May 18;22(5):e1013853. doi: 10.1371/journal.pcbi.1013853 (PMC13211260; doi:10.1371/journal.pcbi.1013853)
Supplement: S3 Text — (PDF) [file pcbi.1013853.s003.pdf]

S3 Text. Robustness testing of the proposed sparse-prior inversion algorithm

To assess the robustness of the proposed sparse-prior inversion against perturbations in the input data, we introduced multiplicative noise into the input vector ***b***:

$$b_i' = b_i\left(1+\eta\xi_i\right), \qquad \xi_i \sim \text{Uniform}[-1,1], \qquad \eta \in [0.05, 0.10].,$$

where η controls the amplitude of noise and ξ_i are independent random variables drawn from a uniform distribution on [−1,1]. For each perturbed vector ***b'***, the following L_1-regularized optimization problem was solved:

$$\min_{x\geq 0} \frac{1}{2} \| Wx - b' \|_2^2 + \delta \| x \|_1 + \sigma \sum_{p=1}^{m} R_p x_p^2 .$$

This procedure was repeated 500 times, where in each trial a new perturbation vector ξ_k was independently sampled and noise amplitude η was randomly selected, generating an ensemble of solutions $\{x^{(k)}\}_{k=1}^{500}$.

Furthermore, the contribution of each flow path was evaluated based on the ensemble distribution, and a stability score was defined as

$$\text{score}_p = \frac{1}{1+\sigma_p / \mu_p},$$

where μ_p and σ_p are the mean and standard deviation of the contribution of path p. A higher score indicates greater robustness of the path flow estimate.

With these elements, Fig S3.1 shows a box plot illustrates the distributions of the top 20 dominant path-flows under 500 noisy tests, along with bar of their stability scores. Narrow interquartile ranges and limited outliers can be observed, and most score values are above 0.9, confirming that the sparse-prior inversion is robust against data perturbations. In particular, as shown in Fig S3.1(c), the two paths with the lowest stability scores (Paths 695 and 86) are visualized to examine their structural characteristics. It can be observed that these two paths share most of their vessel segments and only diverge within two loops including the CoW. Such a high degree of overlap causes strong coupling between the two paths, leading to mutual competition during sparse optimization, which may lower their stability scores under the noisy perturbations.

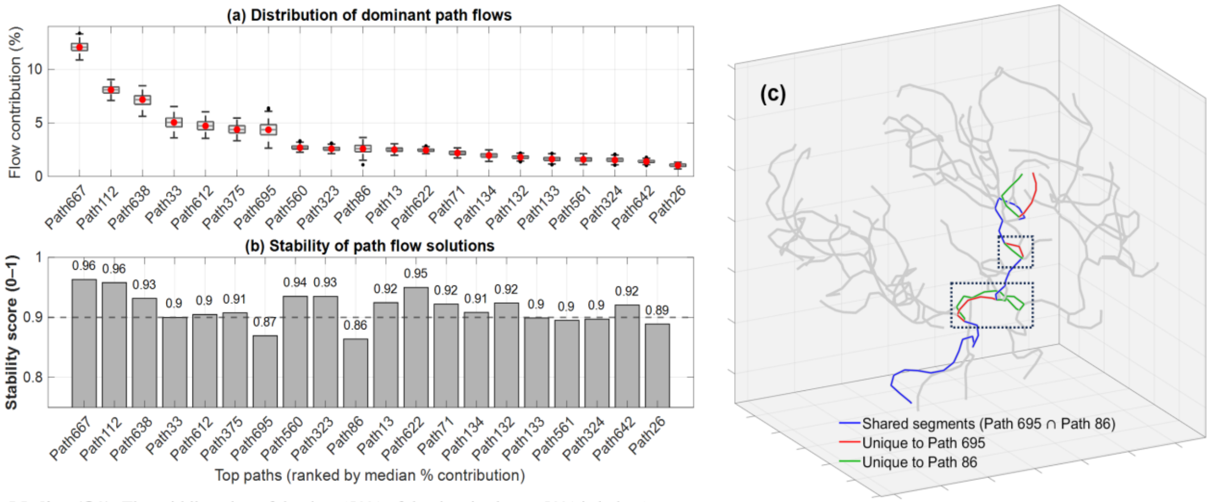

- **Median (Q2)**: The middle value of the data (50% of the data is above, 50% is below).
- **First quartile (Q1, 25th percentile) → Bottom of the box** : 25% of the data are smaller than or equal to this value.
- **Third quartile (Q3, 75th percentile) → Top of the box**: 75% of the data are smaller than or equal to this value.
  Interquartile range (IQR): IQR=Q3−Q1. The box spans this range, which contains the middle 50% of the data.
- **Whiskers**: Extend from Q1 down to the smallest value within Q1−1.5IQR, and from Q3 up to the largest value within Q3+1.5IQR.
- **Outliers (black dots)**: Data points beyond the whiskers.

Fig S3.1. Robustness evaluation of sparse-prior inversion. (a) Flow distribution of the top 20 path across 500 noisy tests, shown as box plots with median values (red filled circles), interquartile ranges (boxes), whiskers, and outliers (black dots). (b) Stability scores. Higher scores indicate smaller variability relative to mean values. (c) Spatial visualization of the two least-stable paths (Paths 695 and 86), which share most of their vessel segments (blue lines) and differ (red and green lines) only within two local loops surrounded by dotted lines.
