# Supplementary material for: Multiscale modeling of blood circulation with cerebral autoregulation and network pathway analysis for hemodynamic redistribution in the vascular network with anatomical variations and stenosis conditions
Source: PLoS Comput Biol. 2026 May 18;22(5):e1013853. doi: 10.1371/journal.pcbi.1013853 (PMC13211260; doi:10.1371/journal.pcbi.1013853)
Supplement: S1 Table — (PDF) [file pcbi.1013853.s004.pdf]

S1 Table. Nomenclature and list of symbols

Symbol	Description	Unit / Eq.
0D Resistance Network (Section 2.1.1)		
R_{ij}	Poiseuille resistance of vessel segment (i, j)	$\mathrm{Pa\,s/m^3}$; Eq. (1)
G_{ij}	Flow conductance, $G_{ij} = 1/R_{ij}$	$\mathrm{m^3/(Pa\,s)}$; Eq. (2)
Q_{ij}	Volume flow rate through segment (i, j)	$\mathrm{m^3/s}$; Eq. (1)
P_i	Nodal pressure at node i	Pa; Eq. (2)
μ	Blood dynamic viscosity	$0.0045\,\mathrm{Pa\,s}$
ρ	Blood density	$1050\,\mathrm{kg/m^3}$
L_{ij}, r_{ij}	Length and mean radius of vessel (i, j)	m
N_{line}	Number of vessel segments	130
N_{node}	Number of nodes in the network	118
S	Number of inflow sources	4
E	Number of outflow sinks	45
St	Estimated Strouhal number of cerebral arteries	$O(10^{-3})$–$O(10^{-2})$
Wo	Estimated Womersley number of cerebral arteries	1.3–2.7
Cardio-Cerebral Coupling (Section 2.1.2, Fig 1c)		
R_{aorta}	Aorta resistance	$\mathrm{Pa\,s/m^3}$
$R_{\mathrm{up},i}$	Aorta → cerebral inlet resistance ($i = 1, \ldots, 4$)	$\mathrm{Pa\,s/m^3}$
R_{arm}	Aorta → Right arm resistance	$\mathrm{Pa\,s/m^3}$
R_{body}	Aorta → Body and left Arm resistance	$\mathrm{Pa\,s/m^3}$
α	Heart pressure scaling factor	–; Eq. (3)
Q_{av}	Aortic valve flow rate	$\mathrm{m^3/s}$; Eq. (3)
Q_{tv}	Tricuspid valve flow rate	$\mathrm{m^3/s}$
Q_{vc}	Flow rate through the vena cava	$\mathrm{m^3/s}$
D_{av}	Aortic valve state function	–; Eq. (3)
P_{lv}, P_{ao}	Left ventricle and aortic pressures	Pa
L_{av}, R_{av}, B_{av}	Valve inertance, viscous, and Bernoulli coefficients	Eq. (3)
$E_{\mathrm{cm,A}}$, $E_{\mathrm{cm,B}}$	Active and passive elastances	Eq. (4)
$e(t)$	Normalised time-varying elastance	–
S_v	Viscoelasticity coefficient of cardiac wall	$\mathrm{Pa\,s/m^3}$
Cerebral Autoregulation Mechanism (Section 2.1.3)		
C_a	Arteriolar compliance	$\mathrm{m^3/Pa}$; Eq. (5)
$C_{a,0}$	Baseline arteriolar compliance	$\mathrm{m^3/Pa}$
ΔC_a^+, ΔC_a^-	Upper/lower compliance bounds	$\mathrm{m^3/Pa}$; Eq. (5)
G_q	Flow feedback gain	–; Eq. (5)
V_{sa}	Arteriolar volume	– (normalised)
f_m	Resistance scaling factor for territory m	–
R_{sa}	Small artery resistance	$\mathrm{Pa\,s/m^3}$
$\bar{R}_{\mathrm{sa}}$	Baseline small artery resistance	$\mathrm{Pa\,s/m^3}$
P_v	Venous pressure	Pa
P_{ic}	Intracranial pressure	Pa
$\bar{q}_m$	Baseline territory flow for territory m	$\mathrm{m^3/s}$; Eq. (5)

Symbol	Description	Unit / Eq.
$\bar{P}_{1,m}$	Baseline distal pressure for territory m	Pa; Eq. (5)
ε_{cam}	CAM convergence tolerance	5×10^{-3}; Eq. (6)
α_{relax}	Factor relaxation coefficient	0.3
1D Blood Flow Model (Section 2.1.4)		
$A(x,t)$	Cross-sectional area	m^2; Eq. (8)
$Q(x,t)$	Volume flow rate	m^3/s; Eq. (8)
$P(x,t)$	Transmural pressure	Pa; Eq. (8)
$A_0(x)$	Reference cross-sectional area	m^2
P_0	Reference pressure (for $A = A_0$)	Pa
P_e	External pressure	Pa
E_s	Young's modulus of vessel wall	0.8 MPa
h_0	Wall thickness	m
K_R	Viscoelastic damping coefficient	$0.5\,\text{s}^{-1}$
c	Moens–Korteweg wave speed	$\approx 7.56\,\text{m/s}$
Δx	Spatial grid spacing	$\leq 0.1\,\text{cm}$
Δt	Time step	$5 \times 10^{-4}\,\text{s}$
τ	Cardiac cycle period	1.0 s
N_{cell}	Spatial cells per vessel	8
C_{CFL}	CFL number	0.3
0D–1D Coupling (Section 2.1.4)		
R_{ij}^{eff}	Effective resistance from 1D area integral	$\text{Pa}\,\text{s/m}^3$; Eq. (9)
γ	Relaxation factor for resistance update	0.5; Eq. (9)
J_n	Outer-loop convergence metric at cycle n	–; Eq. (10)
$\varepsilon_{\text{outer}}$	Outer-loop convergence tolerance	0.01
$N_{\text{outer}}^{\text{max}}$	Maximum outer iterations	20
Stenosis Model (Section 2.2)		
R_s	Stenosis degree (diameter reduction ratio)	%; Eq. (12)
D_0, D_s	Healthy and stenotic diameters	m
A_s	Stenotic cross-sectional area	m^2
L_s	Length of stenotic segment	m
K_t	Turbulence coefficient	1.52; Eq. (11)
K_v	Viscous loss coefficient	–; Eq. (11)
Path-Flow Inversion (Section 2.3)		
$\mathbf{x}$	Path-flow vector (x_p = flow on path p)	m^3/s; Eq. (15)
$\mathbf{W}$	Combined constraint matrix	–; Eq. (14)
$\mathbf{b}$	Observed flow vector	m^3/s; Eq. (14)
λ_1, λ_2	Source/sink constraint weights	–; Eq. (14)
δ	L1 sparsity penalty weight	–; Eq. (15)
σ	Energy (resistance) penalty weight	–; Eq. (15)
R_p	Total resistance along path p	$\text{Pa}\,\text{s/m}^3$
$\theta_{i,j}$	Source-to-sink attribution fraction	–; Eq. (16)
