# Supplementary material for: Multiscale modeling of blood circulation with cerebral autoregulation and network pathway analysis for hemodynamic redistribution in the vascular network with anatomical variations and stenosis conditions
Source: PLoS Comput Biol. 2026 May 18;22(5):e1013853. doi: 10.1371/journal.pcbi.1013853 (PMC13211260; doi:10.1371/journal.pcbi.1013853)
Supplement: Attachment — Submitted filename: review PLOS Computational Biology.pdf [file pcbi.1013853.s005.pdf]

Department of Physics, Faculty of Science
254 Payathai Rd., Wang Mai, Pathumwan,
Bangkok 10310, Thailand

January 13th, 2026

Dear Professor Beni,

I am grateful for an opportunity to review the manuscript “Multiscale modeling of blood circulation with cerebral autoregulation and network pathway analysis for hemodynamic redistribution in the vascular network with anatomical variations and stenosis conditions”. The manuscript describes the development of a mathematical model that represents hemodynamic circulation in a cardio-cerebrovascular network that also integrates cerebral autoregulation mechanism (CAM) under normal physiological condition as well as anatomical variations and progressive pathological stenosis conditions. Blood flow distribution in cerebral arteries with complete Circle of Willis (COW) and two anatomical variations are compared to and found to agree well with those reported from phase contrast magnetic resonance imaging if cerebral autoregulation mechanism is included in the calculation. The results indicate that cerebral autoregulation mechanism is essential in maintaining physiological perfusion. Computed results also show that, during progressive stenosis where there is a transition from the healthy COW to the fetal-type posterior cerebral artery, there is a flow reversal in the anterior communicating artery followed by the activation of the ipsilateral posterior communicating artery in agreement with experiments. Overall, the calculation demonstrates how cerebral vascular network reconfigures collateral pathways when there are structural changes, proposing a mathematical model that can be useful in investigating cerebral flow regulations and functional collaterals in the COW. I personally found the manuscript very stimulating and potentially impactful, and there are a few questions I would like to ask the authors as follows.

1. I am wondering if a few sentences discussing the advantage of choosing to employ Eqs. 3 and 4 (in comparison to other proposed models) could be added. The definitions of Q_{tv} and Q_{vc} as well as the values of the parameters R_{av} and B_{av} should be stated in the manuscript. So does the estimated Strouhal number. An explanation of how the output is distributed into (Q^1_{inlet}, …, Q^S_{inlet}, P^1_{inlet}, …, P^S_{inlet}) would be very nice.

2. I am a little confused about the notation in Eqs 1 and 2. In Eq. 1, Q_{ij} is the flow in the segment connecting the two nodes (i and j). Is Q_i in Eq. 2 a flow at the i node or the ith segment? If a sentence describing one of the rows of the matrices such as Q_2 = …. could be added to the below paragraph, the definition of Q_i can be clarified.

3. An example or an explanation of how the values of weighting factors λ_1 and λ_2 in Eq. 14 allow the flexibility in incorporating certain experimental data would helpful for me to see the complete picture.

4. The comparison to the experimental data including those obtained by Zarrinkoob et al. (2015) and by Zhu et al. (2015) is quite solid. I am, however, wondering if there are other experiments employing phase contrast magnetic resonance imaging that can also be compared to the calculated results (especially for the case of missing A1 segment of anterior cerebral artery (ACA) since, among the 94 subjects in the work of Zarrinkoob et al. (2015), the number of subjects with missing A1 segment of ACA is significantly lower than those with complete COW and those having a fetal-type posterior cerebral artery).

5. There are a lot of symbols and parameters as well as abbreviation within the model. If a list of symbols can be added to Appendix A, it would make the manuscript easier to follow.

I believe I have carefully reviewed and considered the manuscript. Should you need any further assistance, please address me directly at the address indicated in this letter. Thank you so much for allowing me an opportunity to consider an exciting work, and looking forwards to hearing from you.

Sincerely,

Panadda Dechadilok
Department of Physics, Faculty of Science
Chulalongkorn University, Bangkok 10330 Thailand
E-mail : panadda.D@chula.ac.th
(Phone) +66 2 218 7556 (FAX) +66 2 253 1150
