# Supplementary material for: Multiscale modeling of blood circulation with cerebral autoregulation and network pathway analysis for hemodynamic redistribution in the vascular network with anatomical variations and stenosis conditions
Source: PLoS Comput Biol. 2026 May 18;22(5):e1013853. doi: 10.1371/journal.pcbi.1013853 (PMC13211260; doi:10.1371/journal.pcbi.1013853)
Supplement: Attachment — Submitted filename: Response to Reviewers.pdf [file pcbi.1013853.s008.pdf]

Jiawei Liu
Assistant Professor
March, 2026

Dear Editor,

We have completed the revision of the manuscript (PCOMPBIOL-D-25-02693, entitled "Multiscale modeling of blood circulation with cerebral autoregulation and network pathway analysis for hemodynamic redistribution in the vascular network with anatomical variations and stenosis conditions"). Please find attached the updated version of the manuscript and the responses to the reviewers' comments.

We sincerely thank the editor and reviewers for their careful evaluation and valuable comments, which have significantly improved the clarity and presentation of the manuscript.

If further revisions are required, we would like to address them, and we really appreciate your help.

Sincerely Yours,

Jiawei Liu

Point-to-point Response to the Referees

PCOMPBIOL-D-25-02693: Multiscale modeling of blood circulation with cerebral autoregulation and network pathway analysis for hemodynamic redistribution in the vascular network with anatomical variations and stenosis conditions

Response to Reviewer: 1

First of all, we thank you for making valuable comments on our manuscript. By closely following the comments, we have revised our manuscript accordingly. The detailed responses are listed below. We hope that the current revision addresses all your concerns.

Reviewer #1: This is an excellent manuscript that is well written and presented with clarity. The figures are superb and highlight the most important findings. The methods are described with unusual detail and the manuscript advances the field in a useful way. Strengths and weaknesses of the approach are discussed.

Comment 1 (C1.1): The only slight concern is that the authors do not appear to have made their source code freely available. The data are linked through URLs but this reviewer did not find information about the computational code itself.

Response 1 (R1.1): We thank the reviewer for raising this important point regarding code availability.

In the revised submission, the MATLAB source code, input data files, and detailed data descriptions required to reproduce the results are now made publicly available without restriction in a GitHub repository: https://github.com/jw-liu/cerebral-autoregulation-model.

Running the main script (**main_WithCAM_coupled_solver.m**) reproduces the multiscale model with the cerebral autoregulation mechanism (CAM) for all three conditions (Baseline, fetal-type PCA, and hypoplastic ACA) and generates the corresponding comparison figures reported in the manuscript.

Furthermore, an algorithmic pseudocode describing the complete computational workflow has been added and provided as Supporting Information (**S1_Appendix**) to further enhance transparency and reproducibility.

Response to Reviewer: 2

First of all, we thank you for making valuable comments on our manuscript. By closely following the comments, we have revised our manuscript accordingly. The detailed responses are listed below. We hope that the current revision addresses all your concerns.

Reviewer #2:
I am grateful for an opportunity to review the manuscript "Multiscale modeling of blood circulation with cerebral autoregulation and network pathway analysis for hemodynamic redistribution in the vascular network with anatomical variations and stenosis conditions". The manuscript describes the development of a mathematical model that represents hemodynamic circulation in a cardio-cerebrovascular network that also integrates cerebral autoregulation mechanism (CAM) under normal physiological condition as well as anatomical variations and progressive pathological stenosis conditions. Blood flow distribution in cerebral arteries with complete Circle of Willis (COW) and two anatomical variations are compared to and found to agree well with those reported from phase contrast magnetic resonance imaging if cerebral autoregulation mechanism is included in the calculation. The results indicate that cerebral autoregulation mechanism is essential in maintaining physiological perfusion. Computed results also show that, during progressive stenosis where there is a transition from the healthy COW to the fetal-type posterior cerebral artery, there is a flow reversal in the anterior communicating artery followed by the activation of the ipsilateral posterior communicating artery in agreement with experiments. Overall, the calculation demonstrates how cerebral vascular network reconfigures collateral pathways when there are structural changes, proposing a mathematical model that can be useful in investigating cerebral flow regulations and functional collaterals in the COW. I personally found the manuscript very stimulating and potentially impactful, and there are a few questions I would like to ask the authors as follows.

Comment 1 (C2.1): I am wondering if a few sentences discussing the advantage of choosing to employ Eqs. 3 and 4 (in comparison to other proposed models) could be added. The definitions of Qtv and Qvc as well as the values of the parameters Rav and Bav should be stated in the manuscript. So does the estimated Strouhal number. An explanation of how the output is distributed into (1 inlet Q , …, QS inlet1, P inlet , …, S inlet P) would be very nice.

Response 1 (R2.1): We thank the reviewer for this helpful suggestion. In the revised manuscript, we have clarified the motivation for employing the heart model (Eqs.3 and 4), explicitly defined the valve-related variables, reported parameter values and estimated Strouhal number in Supporting Information (**S4_Appendix**), and explained how the cardiac output is distributed to the four cerebral inlets (Figure 1(c)). The main revisions are summarized below.

(a) Advantages of employing Eqs. (3)-(4).

The following text is added to clarify the motivation for employing the time-varying elastance heart model with valve dynamics, originally proposed by Sun et al., 1997 and extended by Liang et al., 2009:

"In this study, the time-varying elastance model and valve dynamics are employed for providing physiologically realistic pulsatile pressure and flow waveforms as inflow into the cerebral vascular network while maintaining low computational cost."

(b) Definitions of Q_{tv}, Q_{vc}, and values of R_{av}, B_{av}, along with the estimated Strouhal number.

The explicit definitions of these variables and parameter values have been added to the nomenclature table (shown in **S4_Appendix**). Following the reviewer's suggestion, we also estimated the Strouhal number characterizing the pulsatile flow regime in the cerebral arteries. The estimated range, together with the corresponding Womersley number, is also reported in **S4_Appendix**.

The following description has been added in the revised manuscript:

" Q_{tv} and Q_{vc} denote the flow rates through the tricuspid valve and the vena cava, respectively. R_{av} represents the viscous resistance of the aortic valve, and B_{av} is the Bernoulli resistance of the valves, which is associated with convective acceleration and dynamic pressure losses."

(c) Distribution of cardiac output to the cerebral inlets.

We clarified this mechanism in the revised Figure 1(c) and its caption as follows:

"(c) A resistance-based coupling scheme between the heart model and the cerebral arterial network. The 0D heart model (Eqs. 3–4) derives the aortic flow $Q_{\mathrm{av}}(t)$ and pressure $P_{\mathrm{ao}}(t)$. These outputs are distributed through an extended resistance network consisting of R_{arm}, R_{body}, and $R_{\mathrm{up},i}$, which connects the aortic arch to the right arm (RArm), the systemic circulation (body + left arm), and the cerebral arterial network (a) via four inlet segments: right internal carotid artery (RICA), right vertebral artery (RVA), left vertebral artery (LVA), and left internal carotid artery (LICA). In this framework, these inlet arteries act as inflow sources of the arterial network (marked by red filled circles in (a)), while the distal terminal nodes correspond to outflow sinks (marked by blue filled circles in (a)) whose outlet pressures P_{outlet} are regulated by CAM through iteratively adjusting distal vascular resistance and compliance. The iterative coupling procedure is described in Section 2.1 and illustrated in Figure 2."

C2.2: I am a little confused about the notation in Eqs 1 and 2. In Eq. 1, Qij is the flow in the segment connecting the two nodes (i and j). Is Qi in Eq. 2 a flow at the i node or the ith segment? If a sentence describing one of the rows of the matrices such as Q2 = …. could be added to the below paragraph, the definition of Qi can be clarified.

R2.2: We are sorry for this ambiguous notation. In Eq. (1), Q_{ij} denotes the volume flow rate through the vessel segment connecting nodes i and j. In contrast, the quantity Q_i

represents the net flow balance at node i, rather than the flow through a specific vessel segment. It corresponds to the sum of flows through all vessel segments connected to node i. To clarify this point, we have revised the manuscript to explicitly state the nodal mass-balance relation as follows:

"The mathematical model employed in this study is based on a network representation of the arterial system, in which each vessel segment is modeled as a resistance, and the flow rate Q across the segment is governed by the pressure drop ΔP according to Poiseuille's law (McConnell and Payne 2017):

$$P_i - P_j = \Delta P_{ij} = Q_{ij} R_{ij}, \quad R_{ij} = \frac{8\mu l_{ij}}{\pi r_{ij}^4}, \quad i \in \{1, \ldots, N_{\text{node}}\}, j \in \mathbf{A}(i). \tag{1}$$

Here, μ is the blood viscosity. Let N_{node} denote the total number of nodes in the vascular network, and $\mathbf{A}(i)$ is the set of nodes directly connected to node i. For any pair (i, j) with $j \in \mathbf{A}(i)$, l_{ij}, r_{ij}, and R_{ij} are respectively the length, radius, and vascular resistance of the segment connecting the two nodes i and j.

Next, we aim to achieve overall pressure equilibrium using iterative computations. The cerebral arterial network (Figure 1(a)) is represented by a nodal resistance network with multiple inlet sources and terminal outlets. With the flow conductance $G_{ij} = 1/R_{ij}$ defined for directly connected node pair (i, j) with $j \in \mathbf{A}(i)$, the pressure distribution across the vascular network is determined by mass conservation at any node k:

$$\sum_{j \in \mathbf{A}(k)} G_{kj}(P_k - P_j) = Q_k,$$

where the net nodal flow Q_k is obtained by summing the flows Q_{kj} through all vessel segments connected to node k as defined in Eq. (1). By separating these connecting nodes into interior and boundary nodes, we obtain

$$\left(\sum_{j \in \mathbf{A}(k)} G_{kj}\right) P_k - \sum_{j \in \mathbf{A}_{\text{int}}(k)} G_{kj} P_j = \sum_{j \in \mathbf{A}_{\text{b}}(k)} G_{kj} P_j + Q_k$$

with

$$\mathbf{A}(k) = \mathbf{A}_{\text{int}}(k) \oplus \mathbf{A}_{\text{b}}(k),$$

where $\mathbf{A}_{\text{int}}(k)$ and $\mathbf{A}_{\text{b}}(k)$ are subsets of $\mathbf{A}(k)$ corresponding to interior and boundary nodes, respectively. It is worth noting that due to conservation of mass, the flow term Q_k should be zero for all nodes other than the inlet and outlet nodes within the network. In this way, the pressure distribution across the entire cerebral vascular network is determined by the mass-balance equation system:

$$\mathbf{BP} = \mathbf{G}_{\text{b}} P_{\text{b}} + Q_{\text{b}}, \tag{2}$$

where the conductance matrix $\mathbf{B}$ is defined as

$$B_{mn}=\begin{cases}\sum_{j\in\mathbf{A}(m)} G_{mj}, & m=n\\ -G_{mn}, & m\neq n, n\in\mathbf{A}_{\text{int}}(m)\\ 0, & \text{otherwise}\end{cases}$$

and the conductance matrix $\mathbf{G}_b$ associated with boundary nodes is defined as

$$(G_b)_{mn}=\begin{cases}G_{mn}, & n\in\mathbf{A}_b(m),\\ 0, & \text{otherwise.}\end{cases}$$

Here, the pressure and flow rate vectors (P_b, Q_b) correspond to the boundary nodes, including S inlets and E outlets as shown in Figure 1(c). The inlet boundary conditions are determined by the upstream resistance network that distributes the cardiac output modeled by the 0D heart model, while the outlet pressure at terminal nodes are regulated by the CAMs, which are introduced in the following subsections."

C2.3: An example or an explanation of how the values of weighting factors 1 in Eq. 14 allow the flexibility in incorporating certain experimental data would helpful for me to see the complete picture.

R2.3: Thanks for this helpful suggestion. In Eq.(14), the weighting factors λ_1 and λ_2 provide flexibility to incorporate experimental measurements with different levels of confidence. In the present study, all segment flows, inflow rates at source nodes, and outflow rates at terminal nodes are derived consistently from the multiscale model, and are therefore treated with equal confidence by setting $\lambda_1 = \lambda_2 = 1$.

In practice, this formulation allows the framework to integrate model predictions and experimental measurements with different levels of confidence. For example, if reliable measurements of inflow rates at the ICA or VA (source nodes) are available, a larger value of λ_1 can be used to enforce closer agreement with these observations. Similarly, if terminal perfusion flows are measured (e.g., from perfusion imaging), λ_2 can be increased to strengthen the sink-node constraints. This flexibility allows the inversion framework to incorporate experimental data of varying reliability and potentially improve the accuracy of the path-flow inversions.

The following description has been added after Eq.(14) in the revised manuscript:

"For example, if reliable measurements of inflow rates at source nodes (e.g., ICA or VA from phase-contrast MRI) are available, a larger value of λ_1 can be used to enforce closer agreement with these observations. "

C2.4: The comparison to the experimental data including those obtained by Zarrinkoob et al. (2015) and by Zhu et al. (2015) is quite solid. I am, however, wondering if there are other experiments employing phase contrast magnetic resonance imaging that can also be compared to the calculated results (especially for the case of missing A1 segment of anterior cerebral artery (ACA) since, among the 94 subjects in the work of

Zarrinkoob et al. (2015), the number of subjects with missing A1 segment of ACA is significantly lower than those with complete COW and those having a fetal-type posterior cerebral artery).

R2.4: Thanks for pointing this out. We have surveyed the available literature for cerebral blood flow measurements. However, to the best of our knowledge, Zarrinkoob et al. (2015) provide the most comprehensive dataset reporting flow distributions across major cerebral arteries within CoW for different anatomical variations, which includes fewer subjects with missing A1 segment of ACA than other cases. Therefore, in the present study we use this dataset as the primary reference for validation.

We agree that additional experimental datasets would be valuable, and will continue to seek and incorporate additional datasets, particularly for the missing ACAA1 case. This will be an important direction for our future work.

C2.5: There are a lot of symbols and parameters as well as abbreviation within the model. If a list of symbols can be added to Appendix A, it would make the manuscript easier to follow.

R2.5: We thank the reviewer for this suggestion. In the revised submission, we have added a comprehensive nomenclature table as **S4_Appendix** that lists the principal symbols, parameters, and abbreviations used in the model together with their equation references.

A reference to this table has been added in Section 2 of the revised manuscript:

"For clarity, a complete list of symbols and abbreviations used in the model is provided in the nomenclature table in Supporting Information (S4_Appendix)."

C2.6: I have some difficulty going through the file artery.csv since there is only one column. If an explanation could be added to this, it would be nice.

R2.6: We apologize for the confusion caused by the format of the original data file.

In the revised submission, the data have been reorganized into structured and well-documented MATLAB (.mat) files to improve clarity, usability, and reproducibility. All data and associated scripts are now publicly available in the GitHub repository: https://github.com/jw-liu/cerebral-autoregulation-model.

Response to Reviewer: 3

First of all, we thank you for making valuable comments on our manuscript. By closely following the comments, we have revised our manuscript carefully. The detailed responses are listed below. We hope that the current revision addresses all your concerns.

Reviewer #3:
The present manuscript provides a multisystems model of the cerebral circulation, including cerebral autoregulation (defined as CAM), a 1D model of the major arteries, and 0D lumped parameter representations of the rest of the circulation. The authors use this framework to understand how anatomical variability and CAM play a role in hemodynamics in the brain, including perfusion.

Overall, the methodology seems innovative. However, this manuscript was incredibly difficult to read, follow, and understand. In particular, the modeling infrastructure was hard to evaluate given all the moving parts that were not clearly linked together. The analysis of "sinks" and "sources," as well as the path analysis, were not clear to this reviewer at all, and hard to evaluate in the results section. The clarity of this manuscript must be improved, otherwise this article's impact cannot be assessed.

1. Section 2.1 (general): It isn't clear how the 0D cerebral resistance terms (eq. (2)) are defined in the larger context of the multiscale model. A schematic showing the exact locations in the circulation where each modeling framework is used is warranted.

   Response 3.1 (R3.1): Thank you for this helpful comment. We agree that the connections between the different components of the multiscale framework were not sufficiently clear in the previous version of the manuscript.
   To improve clarity, Figure 1(c) has been revised to explicitly illustrate the overall coupling structure of the multiscale model. The updated schematic shows the locations where each modeling framework is applied, including the 0D heart model, the 0D resistance network, the cerebral arterial network, and the CAM at terminal nodes. The iterative coupling procedure between these modeling components is described in Section 2.1 and illustrated in Figure 2.
   The description of Figure 1(c) has been revised:

   "(c) A resistance-based coupling scheme between the heart model and the cerebral arterial network. The 0D heart model (Eqs. 3–4) derives the aortic flow $Q_{av}(t)$ and pressure $P_{ao}(t)$. These outputs are distributed through an extended resistance network consisting of R_{arm}, R_{body}, and $R_{up,i}$, which connects the aortic arch to the right arm (RArm), the systemic circulation (body + left arm), and the cerebral arterial network (a) via four inlet segments: right internal carotid artery (RICA), right vertebral artery (RVA), left vertebral artery (LVA), and left internal carotid artery (LICA). In this framework, these

inlet arteries act as inflow sources of the arterial network (marked by red filled circles in (a)), while the distal terminal nodes correspond to outflow sinks (marked by blue filled circles in (a)) whose outlet pressures P_{outlet} are regulated by CAM through iteratively adjusting distal vascular resistance and compliance. The iterative coupling procedure is described in Section 2.1 and illustrated in Figure 2."

The description following Eq.(2) has been added:

"Here, the pressure and flow rate vectors (P_b, Q_b) correspond to the boundary nodes, including S inlets and E outlets as shown in Figure 1(c). The inlet boundary conditions are determined by the upstream resistance network that distributes the cardiac output modeled by the 0D heart model, while the outlet pressure at terminal nodes are regulated by the CAMs, which are introduced in the following subsections."

2. Section 2.2.1, page 5: It isn't clear how the 0D resistance network interactions with the CAM and the 1D network. The use of "sources" and "sinks" in the definition of this model type makes it unclear what the authors mean here. More clarity is needed.

R3.2: We agree that the meanings of "sources" and "sinks" in the previous manuscript were not sufficiently explained.

In the revised manuscript, Figure 1(c) now explicitly illustrates the physical interpretation of these nodes, and the coupling between the 0D resistance network, the 1D cerebral network (Figure 1(a)), and the CAM.

Specifically, the four inlet arteries (RICA, LICA, RVA, and LVA) are treated as source nodes ($S = 4$), representing the blood inflow from the upstream circulation into the cerebral arterial network. The distal terminal nodes correspond to sink nodes ($E = 45$), where blood exits the modeled arterial tree toward the downstream microcirculation. The outlet conditions at these terminal nodes are regulated by the CAM, which iteratively adjusts distal resistance and compliance.

This clarification has been added to the revised manuscript and illustrated in Figure 1(c) and its accompanying description (See **Response R3.1**).

In this work, the terminology "sources" and "sinks" is used to maintain consistent with the formulation of the subsequent path-flow inversion problem, which the cerebral arterial network is represented as a directed flow network. In this formulation, the inlet arteries correspond to inflow sources, and the terminal outlets correspond to sinks. We therefore adopt this terminology from network-flow analysis, rather than conventional physiological inlet and outlet terminology, to match the inversion framework.

3. Section 2.1.2, page 6-7: The authors claim this is a "heart-pulmonary circulation" model, when it appears to just be just a heart model. This is confusing as written.

R3.3: Thank you for pointing out this ambiguity. We agree that the term "heart-pulmonary circulation model" was imprecise and may have caused confusion.

In this study, the model described in Section 2.1.2 is a lumped 0D heart model consisting of four cardiac chambers with time-varying elastance and valve dynamics. The pulmonary circulation is not explicitly represented as a separate vascular network in the present work, but its effects are implicitly incorporated through the model variables and parameters. For example, the pulmonary venous pressure P_{pv} is associated with the flow Q_{mv} across the mitral valve.

To avoid ambiguity, we have revised the corresponding terminology throughout the manuscript and now consistently refer to this component as a "0D heart model", following the formulation described in Liang et al. (2009).

4. Section 2.1.3, page 7: The 0D heart model provides inflow to the 0D vascular network model, thus reducing the true effects of wave propagation once the cerebral 0D and 1D models meet up. This is a limitation of the study and should be contrasted to other studies, e.g., Liang et al. (2009).

R3.4-1: The reviewer correctly points out that, in the present framework, the 0D heart model is connected to the cerebral arterial network through lumped resistance elements rather than through a fully resolved 1D aortic model. As a result, pulse wave propagation along the aorta is not explicitly represented.

In contrast to studies such as Liang et al. (2009), which employs a 1D representation of the aorta connected to the heart, our framework focuses primarily on cerebral flow redistribution and collateral circulation. Therefore, the simplified heart–aorta coupling provides computationally efficient inflow conditions for the cerebral circulation while potentially underestimating detailed pulse waveform effects during aortic transmission. This limitation has now been explicitly acknowledged in the revised manuscript (Section 5, Discussion).

"However, several limitations of the present study should be acknowledged. In the present framework, the 0D heart model is connected to the cerebral arterial network through lumped resistance elements (Figure 1(c)), and thus pulse wave propagation along the aorta is not explicitly resolved, whereas Liang et al. (2009) incorporated a 1D representation of the aorta. Consequently, this study may underestimate detailed waveform distortions during transmission through the aorta, although it still provides adequate inflow conditions for investigating cerebral flow redistribution and collateral circulation. "

Section 2.1.3, page 8: Again, it isn't clear to me how the 0D heart, 0D cerebral circulation, and CAM systems can all be solved without incorporating the 1D solutions. Is the thought here that the 0D/CAM models sets boundary conditions, then the 1D model is run "in isolation" with these boundary conditions fixed?

R3.4-2: The reviewer's understanding of the workflow is essentially correct. In our framework, the 0D heart model, the 0D cerebral resistance network, and the CAM systems are first solved together as a unified 0D system to determine nodal pressures and segment flow rates over one cardiac cycle. These quantities are then used as boundary conditions for the 1D blood flow solver, which computes the spatially resolved flow and cross-sectional area in each vessel segment. The resulting 1D area is subsequently used to update the effective resistances in the 0D network (Eq.9), and the coupled system is iterated until the convergence criterion defined in Eq.(10) is satisfied.

Therefore, the 1D model is not run in isolation, but is coupled to the 0D system through a sequential iterative procedure.

To further improve clarity, the implementation of this coupling procedure are now documented in the pseudocode (**S1_Appendix**, newly added) and in the publicly available GitHub repository (https://github.com/jw-liu/cerebral-autoregulation-model).

In addition, we have revised the description preceding Eq. (10) in Section 2.1.4 to more explicitly describe the sequential coupling between the 0D heart, 0D cerebral circulation, and CAM systems with the 1D solutions:

"The 0D and 1D models are coupled through a sequential iterative procedure (see Figure 2 and S1_Appendix). In each outer cycle, the 0D system including the heart model, the cerebral resistance network, and the CAM, is first solved over one cardiac cycle τ to determine the nodal pressures $P_i(t)$ and flow rates $Q_{ij}(t)$ at each segment. These quantities are then used as boundary conditions for the 1D blood flow solver, which computes the spatially resolved flow $Q(x, t)$ and cross-sectional area $A(x, t)$ within each vessel segment. The resulting 1D solutions are subsequently used to update the effective resistances of the corresponding vessels via Eq. (9). This bidirectional coupling for a cardiac cycle τ is repeated until reaching a quasi-steady state"

5. Section 2.1.4, page 9: The authors now provide more insight into the coupling scheme. The prior statements seem in contrast to the text in this section, since they are claiming the entire system is updated at each time step. I highly recommend providing a pseudocode/algorithm for how each component of the model is updated temporally.

R3.5: Thank you for this helpful suggestion. In the revision, we have added pseudocode describing the temporal update procedure of the multiscale model (**S1_Appendix**).

6. Section 2.2, page 10: This implies that stenoses can only occur prior to the 1D network then, correct?

R3.6: No, stenoses are not limited to occurring only prior to the 1D network. In the proposed multiscale framework, a stenosis located on a given vessel segment is coupled to both the 0D and 1D systems.

In the 0D model, the additional pressure drop across the stenosis is incorporated through the Young–Tsai formulation (Eq.11), which augments the Poiseuille resistance with viscous and inertial components. The resulting effective resistance $R^{\mathrm{eff}}{}_{ij}$ is incorporated into the resistance matrix (Eq.2), allowing the stenosis to influence the global redistribution of flow within the cerebral arterial network.

In the 1D model, the stenosis is treated as a localized geometric constriction within the affected vessel segment by prescribing a reduced reference cross-sectional area $A_{s,0}(x)$ over the stenotic region. This spatially varying geometry directly influences the local hemodynamics, including pressure gradients, wave speed, and flow propagation along the 1D vessel.

Through the iterative coupling procedure, the spatially resolved 1D area field $A_s(x,t)$ is used to update the effective vessel resistances in the 0D network according to Eq.(9). This update reflects both the geometric effect of the stenosis and the flow-dependent variations of cross-sectional area predicted by the 1D solver.

Therefore, the stenosis model affects both the 0D resistance network and the reference cross-sectional area used in the 1D Navier–Stokes solver (Eq.8). To avoid possible ambiguity, we have clarified this point in the revised Section 2.2.

"In the 1D solver, the stenosis is represented as a localized reduction of the reference cross-sectional area within the affected vessel segment, capturing the local hemodynamic effects of the segment narrowing."

7. Section 2.3 (general): I don't understand why the authors need to investigate possible candidate pathways for the model. The boundary conditions, 0D/1D system, all interact together to simulate blood flow. So the path of blood flow should not vary, should it? This section needs stronger justification. In general, it is hard to understand what this analysis is trying to do, or how the authors conduct it.

R3.7: This is an important question. The reviewer is correct that the coupled 0D/1D system already determines the hemodynamic state, including pressures, flow rates, and flow directions in each vessel segment. The path-flow analysis introduced in Section 2.3 is not an alternative simulation method, but rather a post-processing analysis applied to the converged hemodynamic solution.

The motivation for this analysis is that the flow rate at each individual vessel segment alone do not reveal how the perfusion of each downstream territory

(e.g., ACA, MCA, PCA) is assembled from multiple inflow sources (ICAs and VAs). In the complex cerebral network with loops and cross-connections such as the CoW, a given vessel segment may carry blood originating from different sources. Therefore, although the multiscale model determines the flow in each vessel, it does not directly quantify which inflow arteries contribute to a specific territory and through which collateral pathways these contributions are delivered.

The path-flow inversion addresses this problem by decomposing the segment-level flows into source-to-sink pathway contributions, thereby enabling quantitative attribution of regional perfusion to specific inflow arteries.

In addition, although the vascular topology defines a set of possible anatomical paths, only a subset of these paths is consistent with the simulated flow directions under the given physiological condition (baseline or ACAA1-missing). The candidate pathways therefore represent feasible source-to-sink routes that are compatible with the hemodynamic solution from the multiscale model. This filtering step significantly reduces the number of possible paths and ensures that the inversion problem remains computationally tractable.

Overall, this analysis provides additional physiological interpretation beyond the hemodynamic state predicted by 0D/1D model. For example, when reduced perfusion is observed in a specific downstream territory, the path-flow inversion can identify which inflow sources contribute less to that territory and through which collateral pathways the reduction in supply occurs. Such information may provide useful insights for planning bypass surgical strategies under different pathological conditions.

To further clarify this motivation, we have strengthened the explanation of the revised Fig.8, and added the following descriptions at the beginning of Section 2.3:

"In this subsection, we introduce a post-processing analysis methodology applied to the converged hemodynamic solution, which does not alter the simulation results, but instead provides an interpretable decomposition of the simulated blood flow at the segments. It is designed to provide further insight into the computed flow distribution and to quantitatively estimate how blood flow is distributed across multiple candidate vascular paths connecting predefined source and sink nodes. This approach may provide useful information for assessing flow redistribution under different pathological conditions, including potential applications in bypass surgical planning."

and in Section 4.4:

"For example, when evaluating potential bypass strategies for reduced perfusion observed in a specified downstream territory, the path-flow inversion can identify which pathways play a critical role in maintaining perfusion and how surgical modification of the network may redistribute source contributions across territories, thereby helping to restore a physiologically balanced perfusion pattern consistent with the baseline flow

distribution."

8. Section 2.3, page 13: "To further evaluate robustness of this optimization algorithm…" It isn't clear what this "noise" is, or where it is added to the system.
Figure 2: I appreciate that the authors generated a figure, but the model solution itself would benefit from a pseudo-algorithm style explanation.

R3.8: The "noise" refers to multiplicative perturbations applied to the path–flow constraint system ***Wx***=***b*** (Eq.14). Specifically, each entry of the vector **b** is perturbed as

b_i: $b_i'=b_i\cdot(1+\eta\xi)$ with $\xi\sim$Uniform$[-1,1]$ and $\eta\in\{0.05, 0.10\}$,

corresponding to 5–10% perturbations. This simulates measurement uncertainty in the flow data used as input to the optimization problem. The perturbed optimization problem (Eq.15) is solved 500 times to evaluate the stability of the inferred path flows.

Details for this robustness test are provided in Supporting Information (**S3_Appendix**). We have clarified this procedure in Section 2.3 in the revised manuscript.

"To further evaluate robustness of this optimization algorithm, we introduced multiplicative noise into the constraint vector **b**: $\mathbf{b}'=\mathbf{b}\cdot(1+\eta\xi)$, where $\xi\sim$Uniform$[-1,1]$ and $\eta\in\{0.05,0.10\}$ represents a 5–10% perturbation of the flow results from the multiscale model. The perturbed L_1 problem (Eq. (15)) was solved 500 times, each with an independently perturbed input b′, to evaluate the stability of the inferred path flows. The box plot and stability scores are reported in Supporting Information (S3_Appendix)."

Additionally, following the reviewer's suggestion, we have added algorithmic pseudocode describing the main steps of the method in Supporting Information (**S1_Appendix**).

9. Section 2.3, page 13: "Due to the extensive number of vessel segments,…" I acknowledge that there are quite a few components to this modeling study. However, not include specific details as to (i) how many vessels are included and (ii) how many compartments are included (i.e., 1D versus 0D components) make this study difficult to replicate or reproduce. These details are needed, even in a supplement, to ensure that other scientists can carry out a similar study.

R3.9-1: In the revised Section 2.3, we have clarified the main structural characteristics of the vascular network and indicated where the detailed information can be found.

"A summary of the network size and model parameters is provided in Supporting Information (S4_Appendix: Nomenclature table)."

Specifically, the cerebral arterial network used in this study contains 130 vessel segments and 118 nodes, including 4 inflow sources and 45 terminal sink nodes. The model combines a 0D heart model, a 0D resistance network, a 1D blood-flow model for the 130 arterial segments, and 45 terminal CAM compartments.

To improve transparency, these quantities are now explicitly listed in Supporting Information (**S4_Appendix**: Nomenclature table), which summarizes the principal symbols and parameters used in the model.

Furthermore, the geometric data of the vascular network (centerline coordinates, radius, vessel lengths, and connectivity) are provided in the publicly available GitHub repository (https://github.com/jw-liu/cerebral-autoregulation-model). These resources enable other researchers to reproduce the model configuration used in this study.

Figure 3(c): Should we interpret these graphs as the amount of flow expected from the RICA, RVA, LVA, and LICA? This would require particle-level fluid flow, would it not?

R3.9-2: No, the contributions from the RICA, RVA, LVA, and LICA shown in Figure 3(c) are computed from the path-flow inversion (Section 2.3), which can be interpreted as an algebraic decomposition of the steady-state flows resulting from the multiscale model, rather than a particle transport simulation.

Experimental or particle-tracking-based validation of this attribution would be an important direction for future work, but is beyond the scope of the present study.

10. Figure 8: The results in this figure are hard to read and limit the reader's ability to interpret them.

R3.10: We agree that the original Figure 8 was difficult to interpret. In the revised manuscript, Figure 8 has been redesigned to improve readability, with additional explanatory text in the figure caption to interpret the results.

To provide a more intuitive understanding of the simulated flow dynamics, we have also included a new figure (Figure 3) that provides a visualization of blood flow propagation over one cardiac cycle via snapshots at representative time points. In addition, videos illustrating the spatiotemporal flow propagation under all three conditions are also available in the GitHub repository (https://github.com/jw-liu/cerebral-autoregulation-model), offering intuitive visualizations to complement the quantitative results.

11. Section 4.4: The authors are limited in their ability to interpret these results entirely, since parameters outside the CAM module likely contribute to flow dynamics. For example, vascular stiffness plays a major role in pulse wave propagation and flow distribution. This should be highlighted in the limitations.

R3.11: We agree with the reviewer that parameters outside the CAM module may also influence cerebral flow dynamics. In the revised Discussion (Section 5), we have clarified this point in the limitations paragraph.

"In addition, parameters outside the CAM model may also influence cerebral flow dynamics, e.g., vascular stiffness plays an important role in pulse wave propagation and pressure transmission along the arterial tree. In the present model, uniform stiffness parameters (Young's modulus E_s and wall thickness h) are assumed for arteries, whereas in reality arterial stiffness may vary with age, disease state, and anatomical location, which may influence flow redistribution patterns."

12. The imaging data from Zenodo is not found at that link. In addition, the OSF link does not work for this reviewer. Does this include code to replicate the results of the study?

R3.12: We thank the reviewer for pointing this out and apologize for the confusion caused by the broken links. These issues have now been corrected. In the revised submission:

(1) The complete MATLAB source code, input data files, and documentation required to reproduce the results are now publicly available without restriction in the GitHub repository: https://github.com/jw-liu/cerebral-autoregulation-model. Running the provided scripts reproduces the multiscale model with the cerebral autoregulation mechanism (CAM) for all three conditions (baseline, fetal-type PCA, and hypoplastic ACA), and generates the corresponding comparison figures reported in the manuscript.

(2) The CT-based imaging data are publicly available from Ii et al. (2020) via the Zenodo repository (https://zenodo.org/record/3707179), and the link has been corrected in the revised Data Availability Statement. In addition, the processed data used in this study (i.e., GeometryData.mat) are provided in the GitHub repository to improve clarity, usability, and reproducibility of the study.

Minor Comments:

1. Introduction, page 3: Please define the abbreviation "CAM" before using it.
2. Methods, section 2.1: "connected to the heart-pulmonary and peripheral …" should there be a "," not a "-"?
3. Figure 1: The authors include the venous circulation in the schematic of the human head; this should be removed to avoid confusion (since the veins are not included here).
4. Section 2.1.2: The authors refer to Figure 1(d), but there is not a (d) subplot provided.

Response: We thank the reviewer for the careful reading of the manuscript and the helpful suggestions.

(1) The abbreviation CAM (cerebral autoregulation mechanism) has now been defined at its first occurrence in the Introduction.

(2) As noted in our response **R3.3**, we have revised the terminology from "heart-pulmonary model" to "heart model" throughout the manuscript to better reflect the model structure. The sentence in Section 2.1 has been revised accordingly.

(3) The schematic in Figure 1(c) has been redrawn, and the venous circulation has been removed.

(4) The incorrect reference to Figure 1(d) in Section 2.1.2 has been removed.
