# Supplementary material for: Multiscale modeling of blood circulation with cerebral autoregulation and network pathway analysis for hemodynamic redistribution in the vascular network with anatomical variations and stenosis conditions
Source: PLoS Comput Biol. 2026 May 18;22(5):e1013853. doi: 10.1371/journal.pcbi.1013853 (PMC13211260; doi:10.1371/journal.pcbi.1013853)
Supplement: Attachment — Submitted filename: Liu_2026.docx [file pcbi.1013853.s006.docx]

Review of “Multiscale modeling of blood circulation with cerebral autoregulation and network pathway analysis for hemodynamic redistribution in the vascular network with anatomical variations and stenosis conditions” by Liu et al.

**Overview:** The present manuscript provides a multisystems model of the cerebral circulation, including cerebral autoregulation (defined as CAM), a 1D model of the major arteries, and 0D lumped parameter representations of the rest of the circulation. The authors use this framework to understand how anatomical variability and CAM play a role in hemodynamics in the brain, including perfusion.

Overall, the methodology seems innovative. However, this manuscript was incredibly difficult to read, follow, and understand. In particular, the modeling infrastructure was hard to evaluate given all the moving parts that were not clearly linked together. The analysis of “sinks” and “sources,” as well as the path analysis, were not clear to this reviewer at all, and hard to evaluate in the results section. The clarity of this manuscript must be improved, otherwise this article’s impact cannot be assessed.

**Major Comments:**

1. Section 2.1 (general): It isn’t clear how the 0D cerebral resistance terms (eq. (2)) are defined in the larger context of the multiscale model. A schematic showing the exact locations in the circulation where each modeling framework is used is warranted.
2. Section 2.2.1, page 5: It isn’t clear how the 0D resistance network interactions with the CAM and the 1D network. The use of “sources” and “sinks” in the definition of this model type makes it unclear what the authors mean here. More clarity is needed.
3. Section 2.1.2, page 6-7: The authors claim this is a “heart-pulmonary circulation” model, when it appears to just be just a heart model. This is confusing as written.
4. Section 2.1.3, page 7: The 0D heart model provides inflow to the 0D vascular network model, thus reducing the true effects of wave propagation once the cerebral 0D and 1D models meet up. This is a limitation of the study and should be contrasted to other studies, e.g., Liang et al. (2009).
5. Section 2.1.3, page 8: Again, it isn’t clear to me how the 0D heart, 0D cerebral circulation, and CAM systems can all be solved without incorporating the 1D solutions. Is the thought here that the 0D/CAM models sets boundary conditions, then the 1D model is run “in isolation” with these boundary conditions fixed?
6. Section 2.1.4, page 9: The authors now provide more insight into the coupling scheme. The prior statements seem in contrast to the text in this section, since they are claiming the entire system is updated at each time step. I highly recommend providing a pseudocode/algorithm for how each component of the model is updated temporally.
7. Section 2.2, page 10: This implies that stenoses can only occur prior to the 1D network then, correct?
8. Section 2.3 (general): I don’t understand why the authors need to investigate possible candidate pathways for the model. The boundary conditions, 0D/1D system, all interact together to simulate blood flow. So the path of blood flow should not vary, should it? This section needs stronger justification. In general, it is hard to understand what this analysis is trying to do, or how the authors conduct it.
9. Section 2.3, page 13: “To further evaluate robustness of this optimization algorithm…” It isn’t clear what this “noise” is, or where it is added to the system.
10. Figure 2: I appreciate that the authors generated a figure, but the model solution itself would benefit from a pseudo-algorithm style explanation.
11. Section 2.3, page 13: “Due to the extensive number of vessel segments,…” I acknowledge that there are quite a few components to this modeling study. However, not include specific details as to (i) how many vessels are included and (ii) how many compartments are included (i.e., 1D versus 0D components) make this study difficult to replicate or reproduce. These details are needed, even in a supplement, to ensure that other scientists can carry out a similar study.
12. Figure 3(c): Should we interpret these graphs as the amount of flow expected from the RICA, RVA, LVA, and LICA? This would require particle-level fluid flow, would it not?
13. Figure 8: The results in this figure are hard to read and limit the reader’s ability to interpret them.
14. Section 4.4: The authors are limited in their ability to interpret these results entirely, since parameters outside the CAM module likely contribute to flow dynamics. For example, vascular stiffness plays a major role in pulse wave propagation and flow distribution. This should be highlighted in the limitations.
15. The imaging data from Zenodo is not found at that link. In addition, the OSF link does not work for this reviewer. Does this include code to replicate the results of the study?

**Minor Comments:**

1. Introduction, page 3: Please define the abbreviation “CAM” before using it.
2. Methods, section 2.1: “connected to the heart-pulmonary and peripheral …” should there be a “,” not a “-“?
3. Figure 1: The authors include the venous circulation in the schematic of the human head; this should be removed to avoid confusion (since the veins are not included here).
4. Section 2.1.2: The authors refer to Figure 1(d), but there is not a (d) subplot provided.
